# Influence of altitudes and development stages on the chemical composition and antioxidant capacity of Andean blackberries (*Rubus glaucus* Benth)

**DOI:** 10.3389/fnut.2024.1501889

**Published:** 2024-11-06

**Authors:** Mabel Guevara-Terán, Eduardo Tejera, Wilson Vásquez-Castillo, Celestino Santos-Buelga, Ana M. González-Paramás, José M. Alvarez-Suarez

**Affiliations:** ^1^Grupo de Bioquimioinformática, Universidad de Las Américas, Quito, Ecuador; ^2^Grupo de Investigación en Polifenoles, Campus Miguel de Unamuno, Universidad de Salamanca, Salamanca, Spain; ^3^Ingeniería Agroindustrial, Universidad de Las Américas, Quito, Ecuador; ^4^Laboratorio de Investigación en Ingeniería en Alimentos (LabInAli), Departamento de Ingeniería en Alimentos, Colegio de Ciencias e Ingenierías, Universidad San Francisco de Quito USFQ, Quito, Ecuador; ^5^Laboratorio de Bioexploración, Colegio de Ciencias Biológicas y Ambientales, Universidad San Francisco de Quito USFQ, Quito, Ecuador

**Keywords:** Andean blackberry, altitude, Anthocyanins, blackberry chemistry, fruit antioxidants, phenolic and flavonoid content, ripeness and maturity, Rosaceae family

## Abstract

**Background:**

The Andean blackberry (*Rubus glaucus* Benth), locally known as “Mora de Castilla,” is a high-altitude fruit rich in phytochemicals, mainly anthocyanins, with potent antioxidant properties. Although its chemical composition has been studied, the influence of altitude on its phytochemical profile and antioxidant capacity is still unclear. This research aimed to investigate the effects of development stages and altitude on the phytochemical profile and antioxidant activity of this fruit.

**Methods:**

Andean blackberry fruits were collected at different altitudes and development stages in the central Andean region of Ecuador. An hydroalcoholic extraction was used to quantify the compounds and determine the total antioxidant activity, while solid phase extraction (SPE) was performed to separate anthocyanins from other compounds. Ultra-high-performance liquid chromatography coupled to a triple quadrupole mass spectrometer (HPLC-DAD/ESI-MSn) was used to identify anthocyanin and non-anthocyanin phenols, while total antioxidant capacity, total polyphenols, total flavonoid content, and total anthocyanin content were quantified spectrophotometrically.

**Results:**

Flavonols and anthocyanins constitute the majority of the flavonoids identified in the Andean blackberry extracts, both in terms of peak areas and number of identified compounds, followed by ellagic and gallic acid derivatives, as well as phenolic acids, mainly hydroxycinnamic acids. Quercetin was identified as the predominant flavonol in unripe berries, where anthocyanins were not significantly present. On the contrary, in ripe berries, cyanidin and its glycosides stood out as the main anthocyanins and predominant compounds. We observed that in the early stages of ripening, the total polyphenol content predominates in the berries and is mainly responsible for their antioxidant capacity. However, as the fruit ripens, the total anthocyanin content increases, becoming the most prominent bioactive compounds in fully ripe berries.

**Conclusion:**

The results suggest that higher altitude environmental conditions may improve the composition, concentration of phenolic compounds, and antioxidant activity of Andean blackberries. Overall, our findings highlight the high functional value of this fruit, supporting its health-protective effects when consumed regularly, either as fresh fruit or in nutraceutical form.

## 1 Introduction

Berries represent a diverse group of fruits with a wide variety of phytochemical compounds, unique flavor, delicate texture, and attractive color (1). They are distributed across several taxonomic families, with the Rosaceae family being one of the most studied (2). Within this family, the genus *Rubus* stands out, comprising more than 750 species, including blackberries which are grown worldwide (3). Among them is *Rubus glaucus* Benth, also known as the Andean blackberry or “mora de Castilla,” native to the Inter-Andean valleys of Ecuador. This valley is situated between 2,200 and 3,200 m above sea level (m.a.s.l.) (4). The fruits are small drupes measuring around 1 to 2.5 cm in diameter (5, 6) and their color varies from glossy red to dull black during ripening (6, 7). The optimal flavor quality of blackberries is achieved at full ripeness, characterized by reduced firmness and a low organic acid content, alongside elevated levels of sugars and anthocyanins (6, 8). The commercial demand for this species is high, with Andean blackberries being harvested continuously. However, growers suggest that peak production typically occurs during the ripening season from September to November (4). In the markets, blackberries are consumed mainly as fresh fruit or as processed products, such as frozen pulp, jam, juice, wine, or tea (7, 9, 10). Phenology, fruit production, and quality are closely associated with environmental conditions (11). Furthermore, scientific studies have identified several key factors, influencing the chemical composition and biological capacities of berries within the genus *Rubus*. These factors include growing location, plant nutrition, storage conditions, time of harvest, and stage of ripeness (7, 12). In fact, the impact of the ripening process on the bioactive composition and the antioxidant properties of blackberries is well described (13). Previous studies suggested that during ripening, levels of sugars, antioxidant capacity, total soluble solids, and particularly anthocyanin content tend to increase, while pH and titratable acidity generally decrease (10, 14–16). On the other hand, climatic factors such as radiation, temperature, precipitation, and light conditions, which are associated with altitude, play a crucial role in influencing fruit quality and determining the final phenolic profile of the berries (17–19).

Andean blackberries are soft and small berries rich in nutrients, providing a source of vitamins (A and B), minerals (calcium, copper, iron, phosphorus, magnesium, potassium, sodium, selenium, and zinc), and sugars (1, 20). In a recent study by our research group, we demonstrated the involvement of Andean blackberry components in the regulation of genes related to antioxidant and anti-inflammatory responses through their ability to modulate the activation of the NLRP3 inflammasome and autophagy (9). In the same way, many studies have highlighted their significant biological potential in human health. Notably, the Andean blackberry emerges as a natural source of phenolic compounds, mainly represented by flavonoids such as anthocyanins (cyanidin and pelargonidin derivatives), flavonols, and flavanols, followed by phenolic acids like hydroxycinnamic acids (detected as glycosides or ferulic, caffeic, and p-coumaric acid esters) and hydrolyzable tannins (ellagic and gallic acid derivatives) (13, 21, 22). This composition categorizes blackberries as functional food (11).

Indeed, epidemiological studies suggest that regularly consuming Andean blackberries provides health benefits, including reducing the risk of cardiovascular disease, diabetes, and cancer (1, 23). Recent pharmacological investigations have suggested that the positive outcomes observed in the treatment of neurodegenerative diseases could be associated with the anticholinesterase and antioxidant activities of *Rubus* sp. (24, 25). Furthermore, blackberries contribute positively to antimicrobial, anti-inflammatory, antiproliferative, and antioxidant effects (23, 25). Several studies have focused on the nutritional composition and bioactive compound content of blackberries from the *Rubus* genus. However, the influence of altitude gradients and stage of ripeness on the chemical composition and biological capacities of Andean blackberries have seldom been studied (19). Thus, the aim of this study was to evaluate the possible variations in the phenolic compounds profile and antioxidant capacity of wild Andean blackberries, specifically concerning these two variables.

## 2 Materials and methods

### 2.1 Sample collection

Andean blackberries (*R. glaucus* Benth) cv. or “Moras de Castilla” were harvested between August and October 2023. The Tungurahua province was selected for the collection as it accounts for 70% of the national surface area dedicated to the cultivation of this berry (5,000 ha) (4). Two contrasting locations within this province were selected for fruit collection: Santa Lucía—El Triunfo, located at 3,200 m.a.s.l., and Tontapí in Los Andes parish at 2360 m.a.s.l. Access to plant genetic resources was granted by means of the “Framework agreement for access to plant genetic resources: MAATE-DBI-CM-2022-0247” celebrated between the Environment Ministry of Ecuador and the Universidad San Francisco de Quito, Ecuador. The criteria used to select the locations were: (i) the quality and abundance of *R. glaucus* populations, (ii) the fruit production level at three different stages of ripeness within the same plant, and (iii) the altitudinal differences between populations. Prof. Wilson Vázquez (Universidad de Las Americas UDLA, Ecuador) authenticated the specimens using the reference specimens deposited in the UDLA research laboratories.

**Figure 1:**
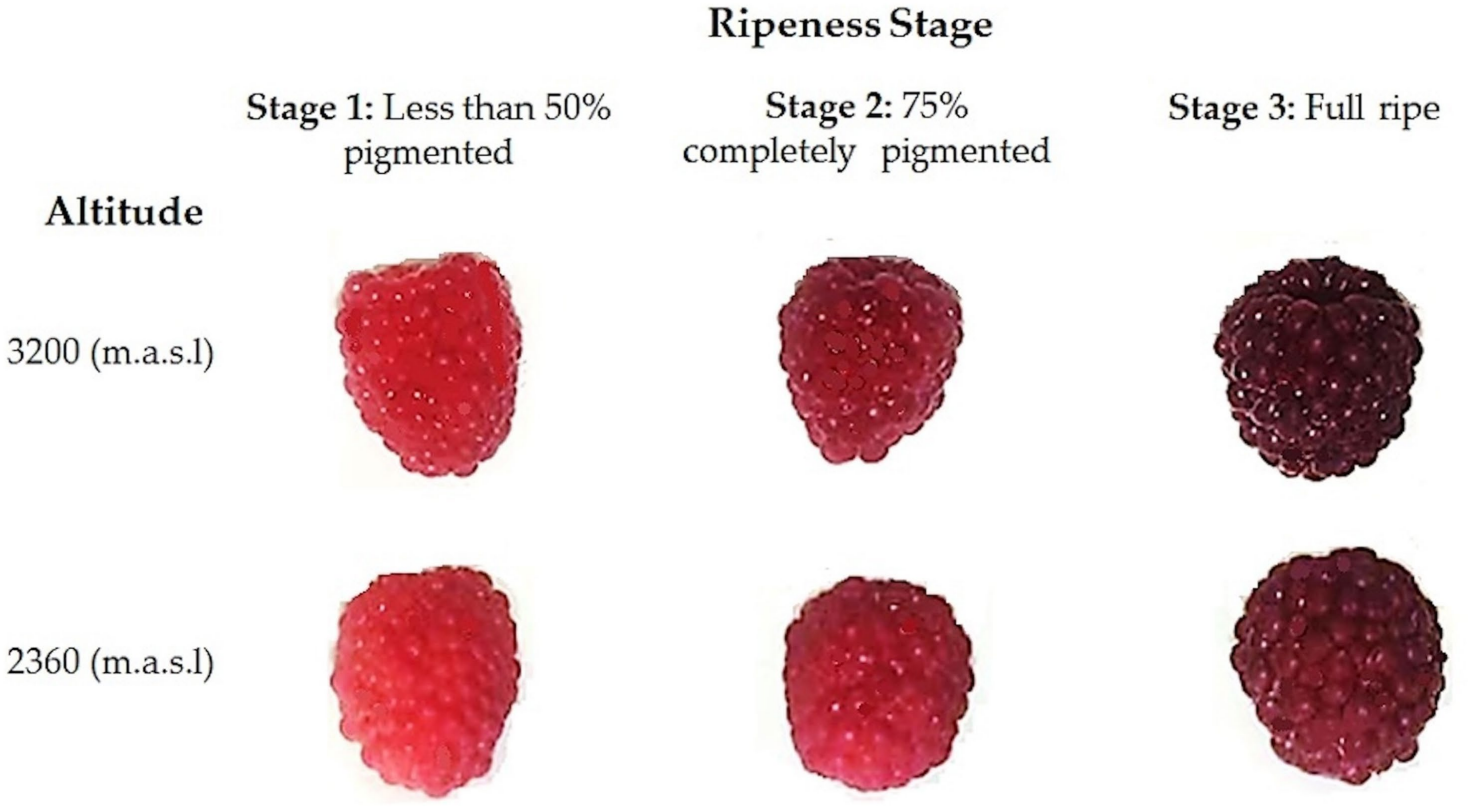
Wild Andean blackberries (*Rubus glaucus* Benth) collected at two different altitudes and at three stages of ripeness, based on their pigmentation.

In each zone, three batches of 200 g of undamaged fruits were randomly collected from different plants in the orchard. The fruits were harvested at three stages of ripeness, based on subjective pigmentation indicators established in the “Normas Técnicas—Instituto Ecuatoriano de Normalización” (NTE INEN) (26). As depicted in Figure 1, color changes during fruit ripening were observed. Less than 50% pigmentation corresponds to the first stage, where the fruits are characterized by predominantly pink drupelets. Fruits with 75% pigmentation correspond to the second stage, where most drupes are deep red with a few purple ones. Finally, the third state (100%), full ripeness, was reached when the fruit displayed entirely purple, almost black drupes (20). No differences in the size or shape of the fruits were noted between the altitudes at the same stage of ripeness.

The harvested fruits were carefully collected and transported to the laboratory. Later, fruits in good condition were selected, and those that suffered any damage during transport (e.g., crushing or draining) were removed. The selected fruits were divided into 6 pools: three ripeness stages for two different altitudes and were immediately frozen at −80°C until further use. The six pools were lyophilized and pulverized using liquid nitrogen and a analytical mill (IKA A11 basic, Germany) until a fine powder was obtained. The samples were kept at a temperature of −80°C until compositional analysis (no more than 2 months from preparation). Table 1 shows the georeferencing of both collection zones located in the province of Tungurahua, Ecuador. Table 2 details the environmental conditions of the collection areas during the months in which the fruits were collected.

**Table 1:** Geographical description of the places where the wild Andean blackberries were collected.

Province	Tungurahua	Tungurahua
Canton	Tisaleo	Patate
Parish	Santa Lucia—El Triunfo	Los Andes (Tontapí hamlet)
Latitude	1°22.183′ S	1°16.052′ S
Length	78°39.545′ W	78°30.298′ W
Altitude (m.a.s.l.)	3,200	2,360

**Table 2:** Environmental conditions of the localities where wild Andean blueberry was collected.

	Temperature (°C)	RH (%)	Dew Point (°C)	Atmospheric pressure (hpa)	Wind (km/h)	Rain (mm year^−1^)
Santa Lucia—El Triunfo						
Average	9.3	85.6	6.9	692.8	3.2	1588.9
Max	3.6	47.0	1.7	689.9	0.00	
Min	17.9	99.0	9.9	697.4	18.4	
Tontapí in Los Andes						
Average	14.5	88.1	12.3	772.5	0.1	1218.0
Max	7.5	35.0	6.7	764.3	0.0	
Min	27.7	99.0	18.2	797.7	1.7	

MAX, maximum, MIN, minimum, RH, Relative humidity.

### 2.2 Preparation of hydroalcoholic extracts

The hydroalcoholic extracts were obtained according to previously described methods (19, 22, 27). Briefly, 1 g of finely powdered samples was extracted using a methanol–water solution (80:20, v/v) acidified with 0.1% hydrochloric acid. The mixture was stirred for 2 h at room temperature, protected from light, and then centrifuged for 10 min at 5,000 rpm (10°C). The resulting supernatant was filtered through a 0.45 μm Minisart syringe filter (Sigma-Aldrich, Whatman, Spain). The solid residue was re-extracted, and supernatants were combined for a total volume of 40 mL. Finally, the hydroalcoholic extracts were stored at −20°C until later analysis.

### 2.3 Total polyphenols, total flavonoid content, and total anthocyanin content

Andean blackberry hydroalcoholic extracts were used for the spectrophotometric analysis of TP, TFC, and TACY. TP was determined using the Folin–Ciocalteu method (19). Results were expressed as milligrams of gallic acid equivalents (GAE) per gram of fresh weight (FW) of fruit (mg GAE/g FW). On the other hand, the aluminum trichloride method (27) was used to determine the TFC, and the results were expressed as milligrams of catechin equivalents (Cateq) per gram of FW of fruit (mg Cateq/g FW). Finally, the TACY was quantified using the pH differential method, and the monomeric anthocyanin pigment concentration was calculated (28). Results were expressed as milligrams of cyanidin-3-glucoside per gram of fresh sample (mg cy-3-glu/g FW).

### 2.4 Polyphenolic profile determination via HPLC-DAD/ESI-MS^n^

For the analysis of phenolic compounds in the blackberry extracts, 1 gram of each previously pulverized fruit pool was extracted using a methanol: water solution (80:20, v/v) acidified with 0.1% hydrochloric acid (10 mL) in an ultrasonic bath for 30 min, followed by centrifugation at 6,700 g for 5 min. The supernatant was collected, and the precipitate was subjected to the same process an additional three times. The resulting hydroalcoholic phases were concentrated until all methanol was removed. Extract purification was performed immediately before HPLC injection by passing 2 mL of the crude extracts through Sep-Pak® C18 reversed-phase solid-phase extraction (SPE) cartridges. The phenolic compounds retained in the cartridge were eluted with a solution of methanol containing 0.1% trifluoroacetic acid (TFA; 95:5) and concentrated to dryness using a rotary evaporator. After evaporation, a final dry product was obtained and stored at 4°C until further analysis. For the analysis of non-anthocyanin phenolics, 3 mg of the dry product from each sample were weighed and dissolved in 1 mL of 0.1% formic acid: acetonitrile (70:30, v/v). This solution was re-dissolved five times before injection into the chromatograph. For anthocyanin analysis, the same procedure was followed, but in this case, the samples were dissolved in 1 mL of 0.1% trifluoroacetic acid (TFA): acetonitrile (75:25, v/v) and analyzed using HPLC-DAD-ESI/MS.

#### 2.4.1 Analysis of non-anthocyanin phenolics

HPLC analysis was conducted using double online detection via diode array spectrophotometry and mass spectrometry (MS). The system comprised a Hewlett-Packard 1,200 chromatograph (Agilent Technologies, Santa Clara, CA, USA) equipped with a binary pump, a diode array detector (DAD), and an HP ChemStation (rev. A.05.04) data processing station (rev. A.05.04). The system was connected to an MS detector API 3200 Qtrap (Applied Biosystems, Darmstadt, Germany) controlled by Analyst 5.1 software. An Agilent Poroshell 120 EC-C18, 2.7 μm (4.6 × 150 mm) column, thermostated at 35°C, was used to separate compounds. The solvents used were: (A) 0.1% formic acid, and (B) acetonitrile. The elution gradient established was isocratic 15% B for 5 min, 15–20% B over 5 min, 20–35% B over 10 min, 35–50% B over 10 min, 50–60% B over 2 min, and isocratic 60% B for 5 min, followed by re-equilibration of the column. The flow rate was set at 0.5 mL/min. The double online detection was performed using the DAD at preferred wavelengths of 280, 330, and 360 nm, and the MS operated in the negative ion mode. Zero air was used as a gas nebulizer (40 psi) and as a turbo gas (400°C, 30 psi) for eluent removal, while nitrogen was used as a curtain gas (10 psi) and collision gas (medium). Both quadrupoles were set at unit resolution, and EMS and EPI analyses were also performed. The EMS parameters were ion spray voltage 4,500 V, DP -50 V, EP -6 V, CE −10 V, and cell exit potential (CXP) -3 V, whereas EPI settings were: DP -50 V, EP -6 V, CE −25 V, and CES 0 V. Spectra were recorded between m/z 100 and m/z 1,400.

#### 2.4.2 Anthocyanin analysis

The separation was carried out on an AQUA (Phenomenex) C18 column (5 μm, 150 mm × 4.6 mm) thermostated at 35°C. The mobile phase consisted of: (A) 0.1% TFA in water and (B) acetonitrile. The elution gradient was programmed as follows: isocratic 10% B for 3 min, 10–15% B over 12 min, isocratic 15% B for 5 min, 15–18% B over 5 min, 18–30% B over 20 min, and 30–35% B over 5 min, followed by re-equilibration to initial solvent conditions. The flow rate was set at 0.5 mL/min. Dual online detection was performed using DAD at a preferred wavelength of 520 nm, alongside MS operating in positive ion mode. Mass spectra were acquired between m/z 100 and m/z 1,500. The MS detector was programmed for consecutive analyses: Enhanced MS (EMS) for high-sensitivity full scans, and Enhanced Product Ion (EPI) for fragmentation patterns of parent ions, using conditions similar to those described in (29). Likewise, the phenolic compounds were characterized based on their UV–Vis absorption spectrum, mass spectrum, and retention time.

### 2.5 Quantification of total antioxidant capacity

The TAC of hydroalcoholic extracts was determined using ABTS^·+^ (2′2-azinebis-3-ethylbenzothiazolin-6-sulfonic acid) radical cation scavenging activity assay (30) and the ferric reducing/antioxidant power (FRAP) method (31). Trolox was used as a standard, and the results were expressed as μmol of Trolox equivalents (TEq) per g of FW of plant (μmol TEq per g FW) for both methods.

### 2.6 Statistical analysis

IBM SPSS Statistics software for Windows version 25.0 (SPSS Inc., Chicago, IL, USA) was used to perform the statistical analysis. Samples were analyzed in triplicate and the results were reported as means with standard deviations (SD). A One-Way ANOVA and Bonferroni *post hoc* tests were applied to compare the data between the different groups. Results were represented by grouped bar graphs along with the standard error. A *p*-value <0.05 was considered statistically significant, while a *p*-value <0.01 was considered highly significant.

## 3 Results

### 3.1 HPLC-DAD/ESI-MS^n^ characterization of phenolic acids, flavonoids, and anthocyanins

**Figure 2:**
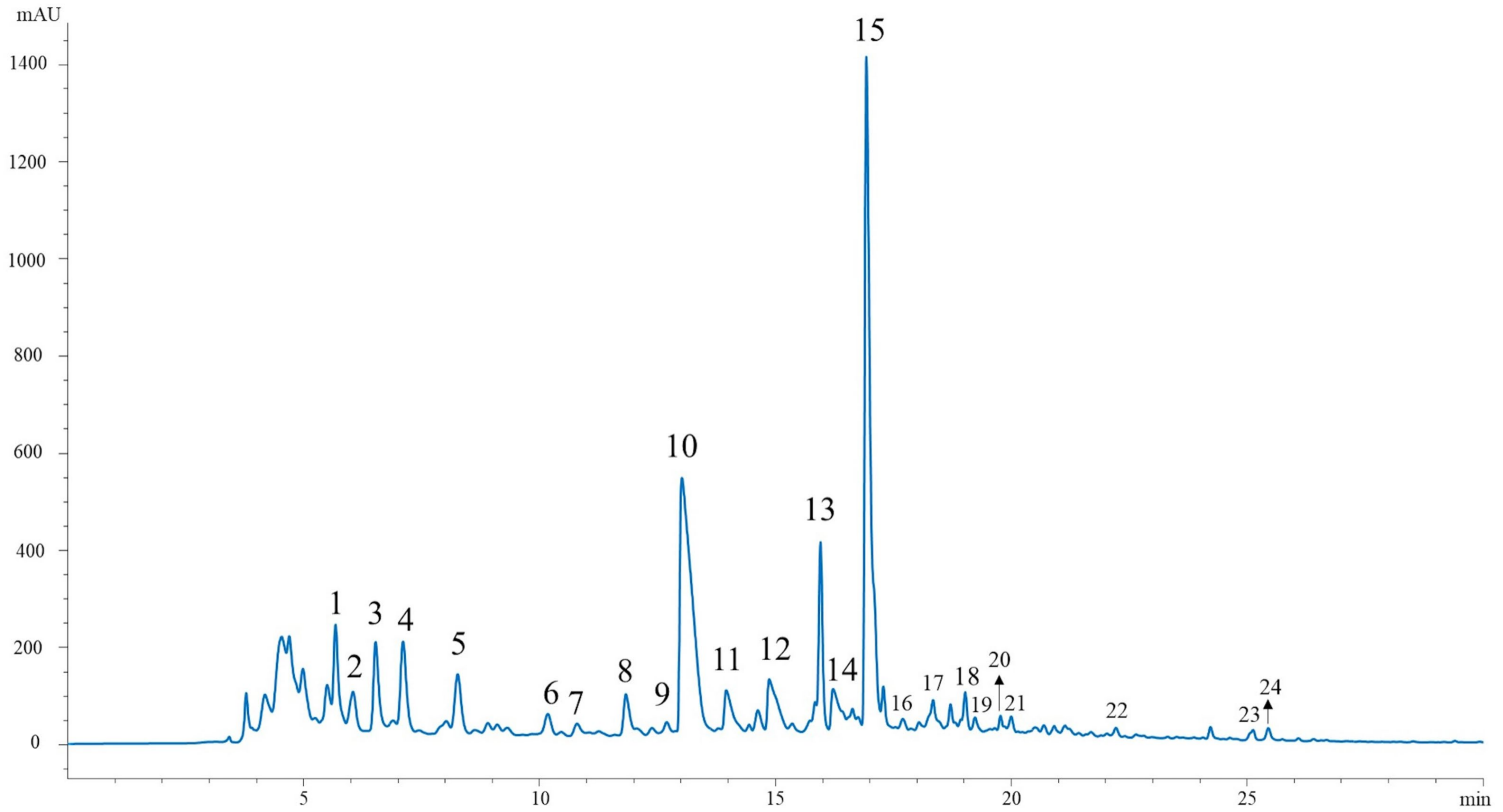
HPLC chromatogram of phenolic acids, ellagitannins derivatives and flavonoids recorded at 330 nm in extracts of Andean blackberry.

**Figure 3:**
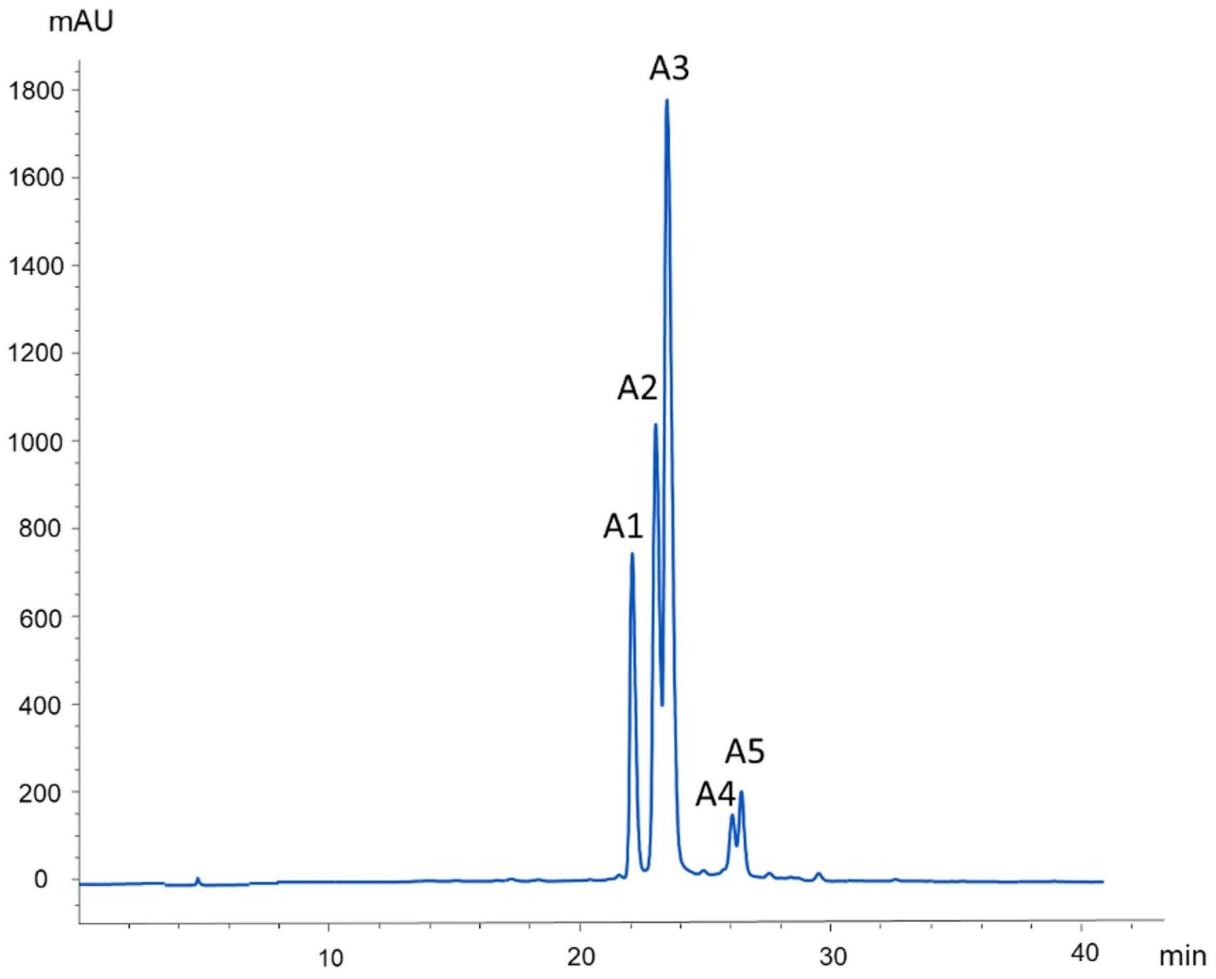
HPLC Chromatogram at 520 nm of anthocyanin extracts from Andean blackberry.

The phytochemical composition of the Andean blackberry collected at three ripening stages and two different altitudes was examined using the HPLC-DAD-ESI/MSn technique. Representative HPLC chromatograms are shown in Figure 2 (*λ* = 330 nm) for phenolic acids, ellagitannin derivatives, and flavonoids, and in Figure 3 (λ = 520 nm) for anthocyanins. Mass spectral data are presented in Tables 3, 4. These tables include (pseudo)molecular ions, MS2 fragmentation patterns, and the tentative identity of the peaks assigned based on these characteristics. The individual phenolic compounds were tentatively identified from their retention time, and UV–Vis and mass spectra. A total of 30 compounds were tentatively identified, corresponding to flavonols (13) and phenolic acids (20), together with ellagic acid derivatives (21) and ellagitannins (20), and anthocyanins (20).

**Table 3:** Mass spectral data and tentative identification of anthocyanins detected in *Rubus glaucus* Benth using HPLC-DAD/ESI-MS^2^.

Peak	RT (min)	[M]^+^ (m/z)	Main MS^2^ product ions (m/z)	Tentative identification
A1	22.1	727	287	Cyanidin-3-O-pentosyl-rutinoside
A2	23.2	449	287	Cyanidin 3-O-glucoside
A3	23.5	595	449, 287	Cyanidin 3-O-rutinoside
A4	26.0	433	271	Pelagonidin-3-O-glucoside
A5	26.5	579	433, 271	Pelagonidin-3-O-rutinoside

**Table 4:** Tentative Identification of non-anthocyanin phenolic compounds in Andean blackberries using HPLC-DAD/ESI-MS^n^.

Peak	RT (min)	Pseudomolecular ion [M-H]^−^ (m/z)	Main MS^2^ product ions (m/z)	Tentative identification
1	5.9	633	301	Galloyl-HHDP glucoside isomer
2	6.2	633	301	Galloyl-HHDP glucoside isomer
3	6.6	935	633, 301	Galloyl-bis-HHDP glucoside isomer
4	7.1	323	161 [138 + 23 uma]	p-hydroxybenzoyl hexoside
5	8.3	325	163, 145	p-coumaroyl hexoside
6	10.2	193	135	Ferulic acid
7	10.9	355	179, 161	Caffeic acid glucuronide
8	11.8	197		Syringic acid
9	12.8	935	467, 301	Galloyl-bis-HHDP glucoside isomer
10	12.9	935		Galloyl-bis-HHDP glucoside isomer
11	13.9	433	301	Ellagic acid pentoside isomer
12	14.9	433	301	Ellagic acid pentoside isomer
13	16.0	609	301	Quercetin rutinoside
14	16.2	301	257, 229, 185	Ellagic acid
15	17.0	477	301, 179, 151	Quercetin glucuronide
		463	301,179, 151	Quercetin glucoside
16	18.1	433	301, 271, 179	Ellagic acid-pentoside isomer
17	18.4	505	463, 301	Quercetin acetylhexoside
18	19.0	433	301, 271, 179	Quercetin pentoside
19	19.3	461	285	Kaempferol glucuronide
20	19.8	447	315, 301	Methylellagic acid pentoside
21	19.9	475	301	Ellagic acid acetylpentoside
22	21.2	463	301	Quercetin hexoside
23	24.4	517	301	Quercetin--malonylpentoside
24	25.5	301		Quercetin

As is characteristic of most species of the *Rubus* genus, the chromatogram showed several peaks corresponding to ellagitannin derivatives (peaks 1–3 and 9–10). All of these were identified by the presence of the fragment ion at m/z 301, which is indicative of ellagic acid. The compounds corresponding to peaks 1 and 2 (Table 3; Figure 2), with an m/z of 633, were presumed to be isomers of galloyl-HHDP glucose. Structurally, this is similar to galloyl-bis-HHDP glucose, but with only one hexahydroxydiphenoyl (HHDP) group attached to the glucose moiety. The observed fragmentation pattern of the pseudomolecular ion at m/z 935, with fragments at m/z 633 and m/z 301, is consistent with the characteristic ellagitannins identified in this and other studies on *Rubus* ellagitannins (32). These peaks could correspond to different ellagitannins such as casuarictin, casuarinin, or potentillin.

For the tentative identification of flavonols (peaks 13, 15, 17–19 and 22–24) the masses of the sugars bound to the aglycons and the specific fragmentation patterns were used. The sugar moieties include hexoses, which are identified by a mass loss of 162 uma (glucose or galactose), pentoses with a mass loss of 132 uma (xylose or arabinose), and glucuronic acid with a characteristic mass loss of 176 uma. These fragment losses correspond to the cleavage of the sugar from the flavonol backbone (33). Quercetin-glucuronide (peak 15) was the main flavonol identified in the Andean blackberry extracts from both altitudinal zones at all three stages of ripeness. According to the presented results, the qualitative phytochemical profile of all extracts was similar regardless of both factors.

### 3.2 Total polyphenols, total flavonoid, and total anthocyanin content

**Figure 4:**
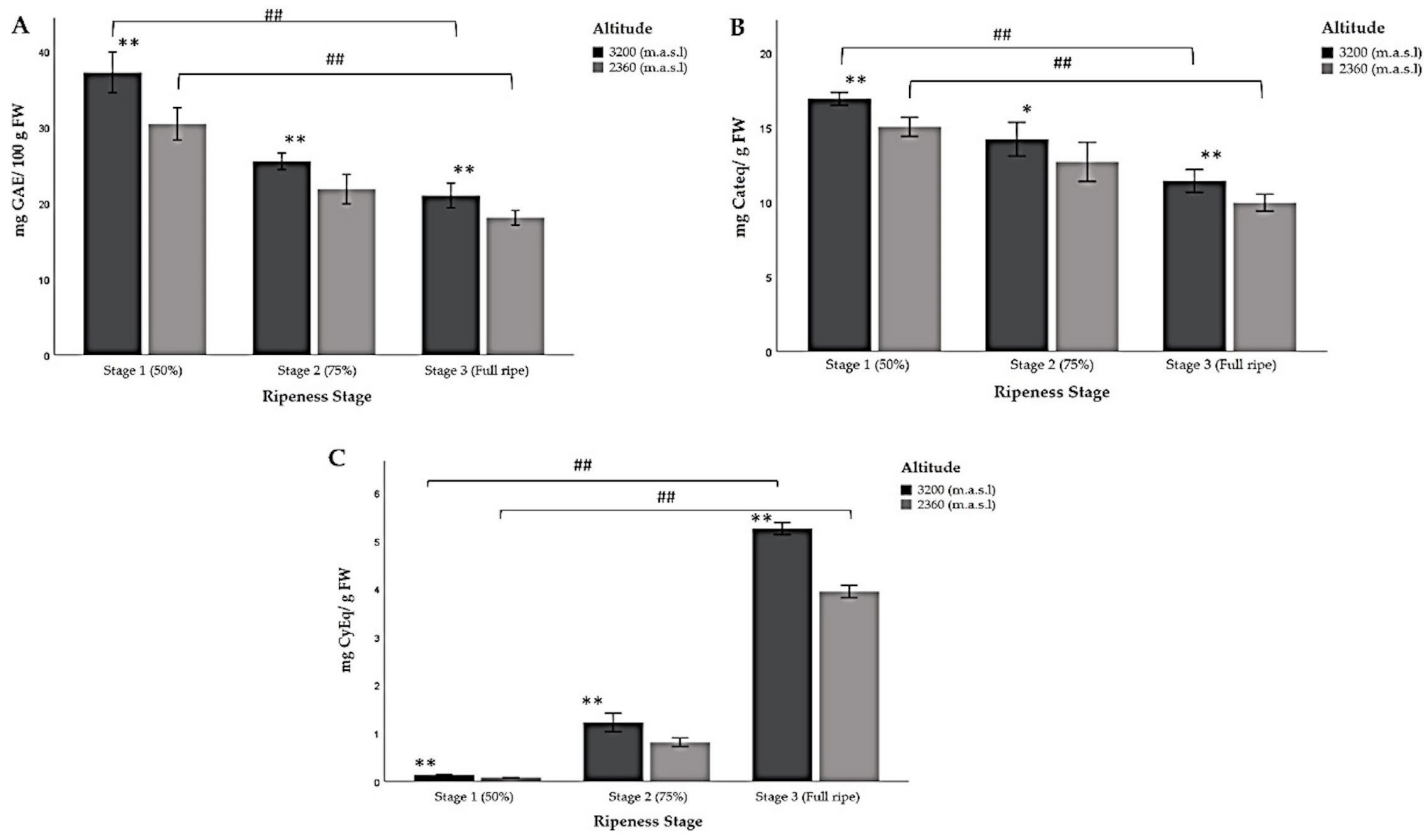
Multiple comparisons of the chemical composition of Andean blackberry (*Rubus glaucus* Benth) at three stages of ripeness (Stage 1: less than 50% pigmented, stage 2: 75% completely pigmented, and stage 3: fully ripe) and at very high (3,200 m.a.s.l.) and high (2,360 m.a.sl) altitudes. (A) Total polyphenols, (B) total flavonoid content, and (C) total anthocyanin content. The results are reported as mean ± SD of three experiments. * *p* < 0.05 and ** *p* < 0.01 are significant differences compared to the lower altitude; < 0.05 and ## *p* < 0.01 are significant differences between the groups of ripeness stages at the lower and higher altitudes.

The highest levels of TP, TFC, and TACY were found at the highest altitude within each stage of ripeness (Table 5; Figure 4). As the fruit ripened, the TP content decreased significantly (Figure 4A). In this regard, the highest measurement was detected at 3,200 m.a.s.l. in ripeness stage 1, when the fruit was immature and their pigmentation was green/reddish. Conversely, the lowest amount was reported at 2360 m.a.s.l., when the fruit was fully ripe (ripeness stage 3).

**Table 5:** Phenolic composition of wild Andean blackberries (*Rubus glaucus* Benth) as affected by site altitude and stage of ripeness.

Parameter	Altitude (m.a.s.l.)	Stage of Ripeness			*p*-value (Stage)
		Stage 1^°^	Stage 2^°^	Stage 3^°^	
TP (mg GAE/100 g FW)^1^	3,200	30.92 ± 0.48	23.06 ± 0.14	18.09 ± 0.66	<0.001
	2,360	26.15 ± 1.81	16.88 ± 0.1	15.60 ± 0.54	<0.001
*p*-value (Altitude)		0.012	<0.001	0.028	
TFC (mg Cateq/g FW) ^1^	3,200	7.15 ± 0.28	6.29 ± 0.29	4.62 ± 0.23	<0.001
	2,360	6.35 ± 0.12	5.01 ± 0.30	4.13 ± 0.21	<0.001
*p*-value (Altitude)		0.011	0.006	0.004	
TACY (mg CyEq/g FW)^1^	3,200	0.13 ± 0.01	1.22 ± 0.09	5.25 ± 0.06	<0.001
	2,360	0.08 ± 0.01	0.81 ± 0.04	3.94 ± 0.06	<0.001
*p*-value (Altitude)		<0.001	0.003	<0.001	

TP, total polyphenols, TFC, total flavonoid content, TACY, total anthocyanins content.

1 Results are expressed as mean ± SD.

° Stage 1: less than 50% pigmented, stage 2: 75% to completely pigmented, and stage 3: fully ripe.

Furthermore, the results showed that the total flavonoid content (TFC) also decreased significantly from the first stage of ripeness (less than 50% pigmented), like the results obtained for TP (Table 5; Figure 4B). The ANOVA indicated that the stage of ripeness significantly affects the TFC (*p* < 0.05; *p* < 0.01). Significant differences were also found between the groups of ripeness stages at the lower and higher altitudes (Table 5; Figure 4).

**Figure 5:**
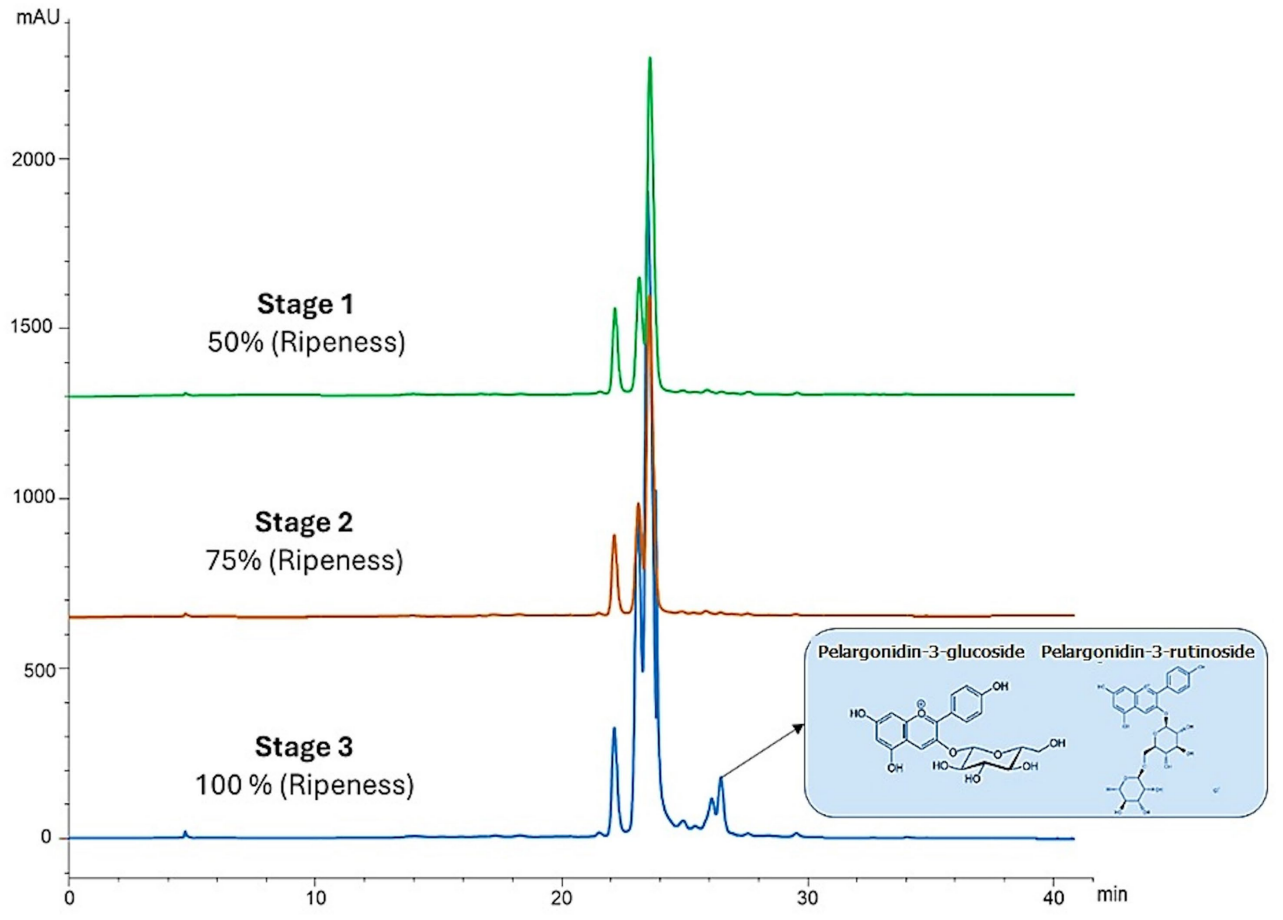
HPLC-DAD chromatograms recorded at 520 nm for Andean blackberries. Comparisons between three stages of ripeness at very high altitude (green: 50% ripeness, orange: 75% ripeness, and blue: 100% ripeness).

As for TACY, the data show a significant increase as the berry’s ripeness increased, unlike TP and TFC (Table 5; Figure 4). The blackberries in the last stage of ripeness (fully ripe) at a very high altitude exhibited the highest TACY content, while the immature fruit (stage 1) harvested at 2,360 m.a.s.l. showed the lowest value. In this context, these types of biomolecules are synthesized during the ripening process. The concentrations for ripeness stages 1 and 2 were low at both altitudes. Moreover, not only were the concentrations of anthocyanins different between ripeness stages but their composition also varied. Figure 5 shows the low concentration of pelargonidin derivatives (pelargonidin-3-glucoside and pelargonidin-3-rutinoside) in stage 3, unlike stages 1 and 2, which lack them. Significant differences were found in all cases (Table 5; Figure 4).

### 3.3 Antioxidant activity of the Andean blackberry (*Rubus glaucus* Benth) relating to stage of ripeness and altitude of the production site

Table 6 shows the results of the TAC of Andean blackberries, evaluated using the ABTS^•+^ radical scavenging assay and the ferric reducing power (FRAP) method. The highest values were obtained using the ABTS method.

**Table 6:** Antioxidant capacity of wild Andean blackberries (*Rubus glaucus* Benth) as affected by altitude and ripeness.

Parameter	Altitude (m.a.s.l.)	Stage of Ripeness			*p*-value (Stages)
		Stage 1^°^	Stage 2^°^	Stage 3^°^	
FRAP (μmol TEq/g FW)^1^	3,200	318.4 ± 4.3	220.1 ± 1.7	127.2 ± 1.1	<0.001
	2,360	260.8 ± 4.4	196.5 ± 2.6	110.0 ± 3.3	<0.001
*p*-value (Altitude)		<0.001	<0.001	0.001	
ABTS (μmol TEq/g FW)^1^	3,200	628.9 ± 5.1	527.1 ± 3.3	414.4 ± 5.7	<0.001
	2,360	537.5 ± 5.1	436.1 ± 12.6	305.1 ± 10.0	<0.001
*p*-value (Altitude)		<0.001	<0.001	<0.001	

FRAP, Ferric reducing/antioxidant power test, ABTS^·+^ (2′2-azinebis-3-ethylbenzothiazolin-6-sulfonic acid) radical cation scavenging activity assay.

1 Results are expressed as mean ± SD.

° Stage 1: less than 50% pigmented, stage 2: 75% to completely pigmented, and stage 3: fully ripe.

**Figure 6:**
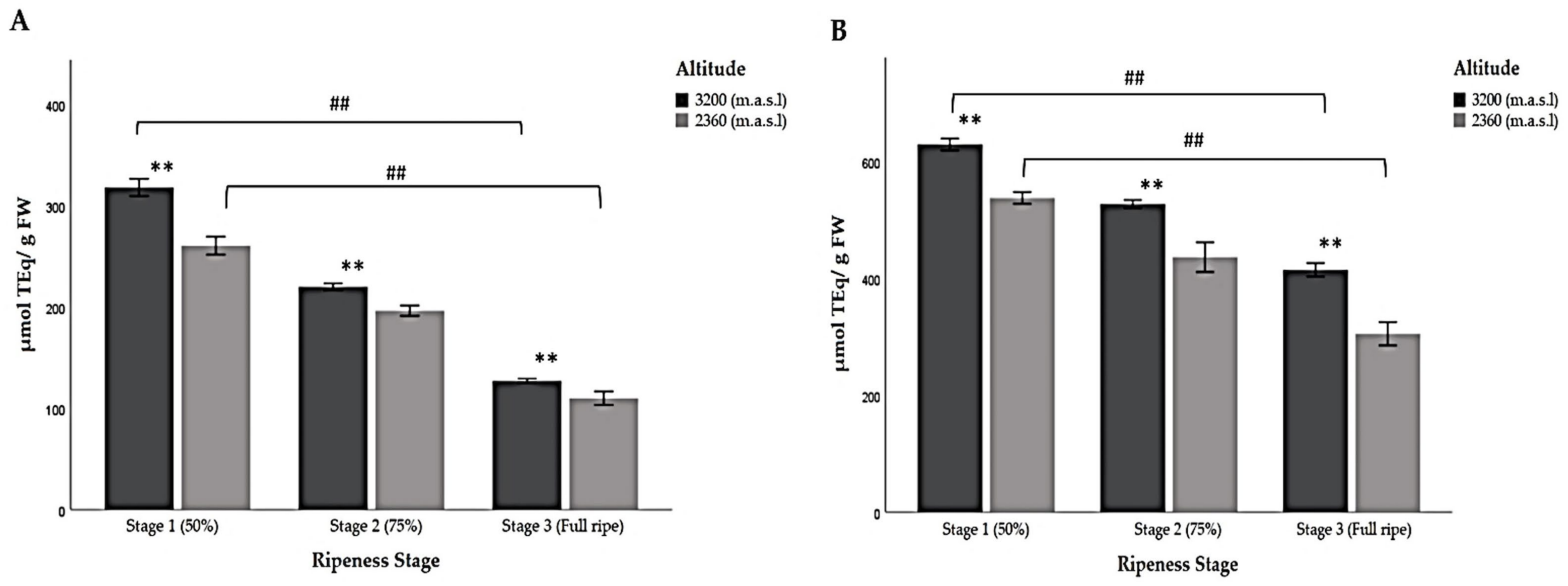
Multiple comparisons of three stages of ripeness in Andean blackberries (*Rubus glaucus* Benth) based on their pigmentation (stage 1: less than 50% pigmented, stage 2: 75% completely pigmented, and stage 3: fully ripe) with respect to very high (3,200 m.a.s.l.) and high (2,360 m.a.sl) altitudes for the fruits’ chemical composition. (A) FRAP assay, and (B) ABTS assay. The results are reported as mean ± SD of three experiments. * *p* < 0.05 and ** *p* < 0.01 are significant differences compared to the lower altitude; # *p* < 0.05 and ## *p* < 0.01 are significant differences between the groups of ripeness stages at the lower and higher altitudes.

The results of the FRAP and ABTS assays show that during ripening, the antioxidant capacity decreased at both altitudes. The highest TAC values were found in samples collected at the higher altitude at ripeness stage 1, while the lowest values were observed in fully ripe berries at the lower altitude in both cases (Table 6; Figure 6). This coincides with the results reported here for TP and TFC (Table 5). Moreover, significant differences were found between stages of ripeness at the higher and lower altitudes when comparing all groups with each other (Table 6).

## 4 Discussion

### 4.1 Phenolic profile of the Andean blackberry (*Rubus glaucus* Benth)

The Andean blackberry (*Rubus glaucus* Benth) is a natural source of phenolic compounds that have been linked to significant health benefits. Among these, flavonoids (including flavanols, flavonols, and anthocyanins) are the predominant class, followed by hydrolyzable tannins (derived from ellagic and gallic acid) and phenolic acids, primarily hydroxycinnamic acids (13, 34). This is consistent with the phenolic profile reported in the present study and in other studies that have examined this species or other blackberry species. That said, one study described the profiles of phenolic compounds in fully mature blackberries (*Rubus* sp.) grown in Boyacá, Colombia, highlighting quercetin derivatives among the identified compounds in *Rubus glaucus* and *Rubus alpinus*. Quercetin glucuronide was predominant in *R. glaucus*, while in *R. alpinus*, quercetin glucoside followed by kaempferol-3-glucoside were the major compounds (35). In another study, the phenolic profiles of Ecuadorian blackberries (*R. glaucus*) and another *Rubus* species (*Rubus adenotrichus*) from Costa Rica were evaluated. The authors tentatively identified two phenolic compounds (quercetin glucuronide and quercetin glucoside), observing that their concentrations were similar across the studied species. These compounds were notably prominent among the identified flavonols, consistent with the findings in our study. Quercetin glucuronide was also identified in *Rubus ulmifolius* Schott harvested from an agricultural property in Alimena, Sicily, Italy, although it did not predominate as in some other species. Notably, epi(catechin), a compound not detected in our study, was found to be the major constituent in this species (14).

As expected, ellagitannins were among the main phenolic compounds found in our Andean blackberry extracts. Ellagitannins are relatively uncommon in our diet but are present in significant amounts in berries of the *Rubus* genus, such as raspberries and blackberries (36, 37). These compounds exhibit a range of beneficial properties including anti-tumorigenic, anti-diabetic, anti-mutagenic, antibacterial, anti-proliferative, and antimycotic effects (38). In addition, they can extend the shelf life of the fruits due to their antimicrobial and antioxidant properties (39). Among the identified ellagitannins, galloyl-bis-HHDP glucose (peak 10) was the principal compound of this group (Figure 3A). It is noteworthy that visualizing isomers of these compounds was challenging due to identical m/z values and similar fragmentation patterns, consistent with findings reported elsewhere (10). For instance, in a study on *R. adenotrichus*, Lambertin C was identified as the major ellagitannin. Conversely, research on *Rubus laciniatus*. Regarding phenolic acids, it is noteworthy that syringic acid (peak 8), ferulic acid (peak 6), caffeic acid (peak 7), and ellagic acid derivatives (peak 11) exhibited low peak areas in this study (Figure 3A). These compounds, particularly ellagic acid, are commonly found in fruits of the *Rubus* genus (40). In recent decades, ellagic acid has garnered significant attention for its ability to regulate cell signaling pathways to mitigate, prevent, or slow down the progression of chronic diseases such as diabetes, neurodegenerative and cardiovascular diseases, and cancer (41). Furthermore, in a recent study by our research group, a hydroalcoholic extract obtained from Andean blackberries was analyzed to determine its polyphenol composition using HPLC-DAD-ESI/MS^n^. The majority of the flavonoid peaks corresponded to the group of flavonols and anthocyanins reported in the present study (9). Among the anthocyanins reported in both studies, cyanidin and its glycosides, like cyanidin 3-*O*-rutinoside (peak 3), were identified as the main components of this group of pigments. In fact, it was found that cyanidin was the predominantly identified form, with only two minor peaks of pelargonidin derivatives detected (peaks 4 and 5; Figure 2). These results are consistent with previous findings. For instance, one study found that cyanidin 3-*O*-rutinoside constituted the major anthocyanin in *Rubus glaucus and Rubus occidentalis*, representing 46 and 39% on average, respectively, of the total anthocyanins. Additionally, similar to us, they also identified cyanidin 3-*O*-glucoside in relatively smaller proportions (5–6%) and pelargonidin 3-*O*-rutinoside in negligible amounts (Figure 6) (42). Regarding Andean blackberries specifically, numerous studies have identified and quantified anthocyanins in this fruit, and the obtained results coincide with the results reported in the present study (7, 8, 34, 35). However, in other species such as, *R. ulmifolius* Schott (Santa Catarina State, Brazil) (16), *R. alpinus* (Boyacá, Colombia) (35), *Rubus croceacanthus*, *Rubus idaeus*, and *Rubus sieboldii* (Okinawa, Japan) (43), cyanidin 3-*O*-glucoside and pelagonidin-3-rutinoside were identified as the main anthocyanins. This variation could be a consequence of agronomic, genetic, and geographical factors (44).

### 4.2 Total polyphenols, total flavonoid, and total anthocyanin content

Few studies have evaluated the influence of altitude and ripeness of the fruit on the physical–chemical composition and antioxidant capacity of the Andean blackberry (*R. glaucus* Benth). According to the results, this fruit is an important natural source of bioactive compounds with relevant antioxidant capacities.

The Folin–Ciocalteu (FC) assay was used to measure the TP of the samples as a specific determination of polyphenols (30). The results were interpreted according to the suggestion of several investigations, which agree that the FC reagent reacts not only with phenolic compounds but also with some reducing sugars, amino acids, vitamins, peptides, and other compounds present in the samples (45, 46). The results determined that altitude is an important factor: the higher the altitude, the greater the TP (Table 5; Figure 4A). Similar results have been previously reported, such as in studies into the content of polyphenols in 10 blackberry cultivars under tropical high-altitude conditions, where authors found variations in the composition of polyphenolic compounds between the cultivars being studied. Additionally, the concentration of these compounds was higher when compared to cultivars grown at lower altitudes (46). Meanwhile, another study on berries determined that altitude significantly influences the concentration of individual phenolic compounds (47). Studies on *Rubus fruticosus* (48) and *Rubus idaeus* (49, 50) reported a high concentration of ellagic acid relative to other phenolic components with increasing altitude, which could be related to the environmental conditions of the cultivation area (46, 50). The combination of extreme environmental conditions and high UV radiation positively influences the reducing capacity of the fruit. This corroborates the results obtained in our study (Table 2). The higher the altitude, the lower the attenuation of the sun’s rays. In this sense, UV radiation tends to increase and temperature to decrease (50). Furthermore, the obtained results showed a significant decline in TP, from ripening (50% pigmentation) to fully ripe fruit at both altitudes (Figure 4A). Previous research has evaluated the reducing capacity of four fruits: black raspberry (*R. occidentalis* L.), blackberry (*Rubus* sp.), red raspberry (*R.idaeus* L.), and strawberry (*Fragaria × ananassa* D.). In both strawberries and blackberries, the TP markedly decreased as the fruit matured from being green to fully ripe (51). This observation is consistent with the results presented here (Table 5; Figure 4A). During development and ripening, substantial shifts occur in the relative contributions of the different classes of compounds (52, 53). In this regard, the differences observed in the three ripening stages across two altitudes could be attributed not only to the total polyphenol content but also to the qualitative phenolic composition. In fact, most phenolic compounds decrease as the fruit ripens, while anthocyanins increase significantly. This finding was consistent with results from red raspberries grown in Finland, where quercetin concentration was remarkably lower when TP was highest (11).

The results demonstrated that the TFC was also influenced by both the stage of ripeness and the altitude of the production sites where the fruits were collected (Table 5; Figure 4B). Similar findings were obtained in a previous study on *Vaccinium floribundum* Kunth, where Andean blueberries collected at higher altitudes exhibited higher concentrations. As the berries began to ripen, the concentration of TP, TFC, and antioxidant capacity decreased (7). Numerous studies have discovered differences in the composition and concentration of individual phenolic contents in berries due to several environmental factors (52). In a recent evaluation of the chemical composition of wild Andean blueberries, a positive correlation was observed between high TFC and altitude. The higher incidence of solar radiation at elevated altitudes led to increased levels of flavonoids, such as quercetin glycosides (7). These variables in turn play an important role in berry ripening (11). Another study examined the chemical composition of raspberry (*R. idaeus* L.) collected from various locations across 17 districts in Lithuania. They reported that environmental, developmental, phenotypic, and genetic factors had a significant effect on the content of quercetin (52).

During the ripening process of berries, many complex biochemical changes take place, such as sweetening, enlargement, and softening. Among these changes is the alteration in fruit pigmentation. Initially, carotenoids, chlorophylls, and flavonols are the predominant pigments, imparting a green color to the fruit in the early stages of maturation. As ripening progresses, the color of the fruit changes from green to pink. This color change is attributed to the degradation of these pigments and the subsequent accumulation of anthocyanins (54, 55). The findings presented in this study corroborate this process, as evidenced by the increase in total anthocyanin content during fruit ripening, which is reflected in the observed color change (Figure 1). Flavonoids can be accumulated in different organs or parts of plants, where they play several important roles. Previous studies have reported that the skin of some berries is the principal area where anthocyanins are present (56, 57). The anthocyanin profile changes as the berries begin to ripen. Studies on raspberries (*R. idaeus*) have indicated that some anthocyanins, like cyanidin-3-glucoside, are already present in unripe (green) berries. However, other anthocyanins, such as cyanidin glucosylrutinoside, cyanidin sophoroside, and pelargonidin glucosylrutinoside, appear only when the red color of the fruit develops. Scientists have also determined that the dominant anthocyanins in this fruit are derived from cyanidin and pelargonidin and differ only in their sugar moieties (58). In fact, previous research on the same species determined that cyanidin and pelargonidin derivatives were the main anthocyanins identified in fully ripe fruits. However, in immature or almost ripe fruits, levels of pelargonidin were not detected (7, 35). This statement is consistent with our results (Table 3; Figure 2). Furthermore, other authors have analyzed the expression of anthocyanin pathway genes and determined that glucosyl transferases were upregulated at the end of the ripening process and are independent of other anthocyanin biosynthesis genes. When glucosyl transferase expression was inhibited, anthocyanin accumulation in the fruit was adversely affected (59, 60).

Focusing now on anthocyanin content, it was found that the higher the altitude, the higher the TACY (Table 5; Figure 4C). This is because as previously mentioned, altitude and environmental conditions at each production site influence the chemical composition of Andean blackberries, including anthocyanin content. Our results partially agree with those reported in a study conducted in Bulgaria, where mature *R. idaeus* L. fruits were collected from seven areas at different altitudes. It was determined that the anthocyanin concentration (TACY) at the highest altitude (1,804 m.a.s.l.) was 4-fold higher than the concentration reported at the lowest altitude (171 m.a.s.l) (54). Temperature, particularly daytime temperature, is a poorly recognized but essential environmental factor in the accumulation of anthocyanins in berries. This factor is closely related to altitude and also influences ripening (50, 61).

### 4.3 Antioxidant activity of the Andean blackberry (*Rubus glaucus* Benth) relating to stage of ripeness and altitude of the production site

Natural antioxidants in fruits, vegetables, and medicinal plants arouse great interest among consumers and scientists (28, 52). The various antioxidant mechanisms of berries may be attributed to the presence of compounds with metal-chelating capacity, effectiveness as hydroxyl radical scavengers, and their strong hydrogen-donating abilities (48). These fruits are effective inhibitors of human low-density lipoprotein oxidation, which may have potential health benefits, with the chemical composition of the fruit playing an important role in this effect (51).

Berries have shown a positive and significant relationship between their phenolic compounds and antioxidant activity. The concentration and composition of polyphenols can vary in response to oxidative stress, which may cause the antioxidant capacity to decrease or increase (5). Previous research has determined that altitude and climatic conditions at the production site can influence antioxidant capacity (5). Scientists studying strawberries (57) and raspberries (62) have observed an increase in the total polyphenol content and antioxidant capacity when the altitude (5) and temperature of the area increased (61). Our results demonstrate that antioxidant capacity also varied according to the ripeness stage of the fruits (Table 6; Figure 6). Previous studies found a linear relationship between antioxidant capacity and anthocyanin or total phenolic content in ripe berries (48). Additionally, they determined that the phenolic composition, anthocyanin, and antioxidant capacities of raspberries, strawberries, and lowbush and highbush blueberries changed during ripeness. The antioxidant activity varied among fruit extracts, with the highest activity observed in blackberries (51). Results from another studies revealed similar variations in the antioxidant activity of red fruit extracts, which may be attributed to the individual actions of the compounds contained in the extracts (57, 62). This is corroborated by a study into *R. idaeus*, which identified cyanidin anthocyanins, ellagitannins (sanguiin H6 and lambertianin C), and proanthocyanidins as the compounds with the greatest influence on antioxidant activity (62). It is well described that anthocyanins increase as the fruit ripens. However, little is known about polyphenols, such as ellagitannins and proanthocyanidins. The study concluded that, unlike anthocyanins, these compounds strongly decrease during berry ripening, but still contribute to 30–60% of the total antioxidant activity (58). Other compounds such as ellagic acid, an important phytochemical, and flavonols (kaempferol and quercetin), although present in lower concentrations, exhibit antioxidant activity and play an important role in the protective effects of berries and vegetables (50, 61). In addition, these compounds have been reported to provide protection against certain types of cancer and viral infections (63, 64). Therefore, the diverse range of bioactive components present in fruits, vegetables, and medicinal and aromatic plants contribute unequally to their total antioxidant activity. Significant shifts in the relative contributions of different compound classes have been observed during the development and ripening of berries. However, these changes cannot be fully detected by a general determination of the total antioxidant activity, so more exhaustive study is required.

## 5 Conclusion

The chemical composition and antioxidant capacity of Andean blackberries (*Rubus glaucus* Benth) are significantly influenced by factors such as altitude and ripening stage. Our findings demonstrate that blackberries harvested at higher altitudes exhibit higher concentrations of key bioactive compounds, regardless of their ripening stage. Notably, the early ripening stages presented the highest levels of total polyphenols, flavonoids, and antioxidant capacity, while the anthocyanin content was most pronounced at full ripeness. These results suggest that the geographical and climatic conditions associated with altitude play a crucial role in modulating the ripening process, nutritional content, and phenolic profile of the blackberries. This knowledge is not only pertinent for optimizing harvest timing based on intended use but also underscores the nutritional value of fully ripe Andean blackberries, which are rich in bioactive compounds and can offer significant health benefits when incorporated into the diet. While immature blackberries are not suitable for direct consumption, their high content of bioactive compounds makes them an excellent raw material for nutraceutical applications. When processed into extracts, these fruits may provide potential health benefits. Collectively, these findings highlight the importance of Andean blackberries in both the food and nutraceutical industries, offering promising opportunities for the development of products with antioxidant properties and other functional benefits.

## Data Availability

The original contributions presented in the study are included in the article/supplementary material, further inquiries can be directed to the corresponding authors.
